# Efficacy of Providing the PI3K p110α Inhibitor BYL719 (Alpelisib) to Middle-Aged Mice in Their Diet

**DOI:** 10.3390/biom11020150

**Published:** 2021-01-25

**Authors:** Christopher P. Hedges, Jordi Boix, Jagdish K. Jaiswal, Bhoopika Shetty, Peter R. Shepherd, Troy L. Merry

**Affiliations:** 1 Discipline of Nutrition, School of Medical Sciences, University of Auckland, Auckland 1023, New Zealand; c.hedges@auckland.ac.nz (C.P.H.); bshe380@aucklanduni.ac.nz (B.S.); 2 Maurice Wilkins Centre for Molecular Biodiscovery, University of Auckland, Auckland 1023, New Zealand; j.jaiswal@auckland.ac.nz (J.K.J.); peter.shepherd@auckland.ac.nz (P.R.S.); 3 Centre for Brain Research, University of Auckland, Auckland 1023, New Zealand; j.boix-i-coll@auckland.ac.nz; 4 Auckland Cancer Society Research Centre, University of Auckland, Auckland 1023, New Zealand; 5 Molecular Medicine and Pathology, School of Medical Sciences, University of Auckland, Auckland 1023, New Zealand

**Keywords:** insulin signaling, aging, BYL-719, glucose tolerance, pharmacokinetics

## Abstract

BYL719 (alpelisib) is a small molecule inhibitor of PI3K p110α developed for cancer therapy. Targeted suppression of PI3K has led to lifespan extension in rodents and model organisms. If PI3K inhibitors are to be considered as an aging therapeutic, it is important to understand the potential consequences of long-term exposure, and the most practical way to achieve this is through diet administration. Here, we investigated the pharmacokinetics of BYL719 delivered in diet and the efficacy of BYL719 to suppress insulin signaling when administered in the diet of 8-month-old male and female mice. Compared to oral gavage, diet incorporation resulted in a lower peak plasma BYL719 (3.6 vs. 9.2 μM) concentration but similar half-life (~1.5 h). Consuming BYL719 resulted in decreased insulin signaling in liver and muscle within 72 h, and mice still showed impaired glucose tolerance and insulin sensitivity following 6 weeks of access to a diet containing 0.3 g/kg BYL719. However, consuming BYL719 did not affect food intake, body mass, muscle function (rotarod and hang time performance) or cognitive behaviors. This provides evidence that BYL719 has long-term efficacy without major toxicity or side effects, and suggests that administering BYL719 in diet is suitable for studying the effect of pharmacological suppression of PI3K p110α on aging and metabolic function.

## 1. Introduction

Phosphoinositol-3-kinases (PI3Ks) are a family of enzymes that participate in the regulation of cellular glucose metabolism, proliferation and survival, through the generation of the lipid signaling molecule phosphoinositide-(3,4,5)-triphosphate (PIP3) by the phosphorylation of phosphoinositide-(3,4)-biphosphate (PIP2) [1]. In particular, the alpha isoform of Class II PI3K (p110α) plays an important role in insulin-stimulated glucose uptake and cell growth [2]. Accordingly, mutations in the gene encoding p110α (*PIK3CA*) and the phosphatase enzyme PTEN which dephosphorylates PIP3 back to PIP2 [3] are found in many cancers. Because of the well-described role of PI3K in the development of cancers, there has been great interest amongst the oncology field in developing small molecule inhibitors of PI3K [4,5].

The leading clinical small molecule PI3K p110α inhibitor is BYL719 (tradename alpelisib by Novartis), which was approved by the FDA in 2019 for the treatment of PIK3CA-mutated advanced or metastatic breast cancer in combination with fulvestrant, and is also a promising therapy for patients with PIK3CA-related overgrowth syndrome [6]. However, the clinical potential of BYL719 and other related PI3K inhibitors may be broader-reaching than initially considered. In *C. elegans*, knockdown of *age-1*, an ortholog of mammalian PI3K, dramatically extends lifespan leading to suggestions that targeted suppression of the PI3K signaling pathway may have promise as a therapeutic for age-related disorders [7,8]. Two of the primary ligands that activate PI3K are insulin and IGF-1, and some subsequent studies in rodents have shown that partial genetic global or tissue specific suppression of the insulin/IGF-1 signaling pathway can extend lifespan [9,10,11,12].

Consistent with observations that lowering insulin/IGF-1 signaling can also extend healthspan as well as lifespan in rodents [9,10,11,12], both genetic and pharmacological inhibition of PI3K has been reported to reduce adiposity and improve the metabolic function of rhesus monkeys and high calorie-diet fed mice [13,14]. These findings appear to be somewhat paradoxical given PI3Ks role in insulin signaling, and observations that inhibition of PI3K can also result in as insulin resistance, glucose intolerance and diabetes-like phenotypes [15,16]. Indeed, our group has previously shown that the acute treatment of mice with a variety of pan- and isoform-specific PI3K inhibitors causes pronounced impairments in insulin sensitivity and glucose tolerance [17]; however, extended (22 d) treatment with the same inhibitors in young (4–6 week old) mice via daily intraperitoneal (i.p.) injection resulted in only mild, if any, impairment in insulin sensitivity [18]. Therefore, in addition to level of suppression, exposure time likely contributes to determining the effect of PI3K inhibitors on metabolic regulation.

If PI3K inhibitors are to be considered as an aging or metabolic therapeutic, it is important to understand the potential consequences of long-term exposure, independent of pre-existing diseases or co-treatments (such as additional cancer therapeutics). BYL719 is orally available, and in the majority of rodent studies it is administered via a once-daily oral gavage or i.p. injection [19,20]. However, this is not feasible for long-term aging or metabolic disease studies where treatment time may be in excess of 1 year. In such studies, the common drug delivery method is through supplementation in the diet [21,22,23]. Therefore, we assessed the pharmacokinetics and efficacy (i.e., the ability to maintain PI3K inhibition following chronic exposure) of longer-term treatment with the PI3K p110α selective inhibitor BYL719 when administered in the diet of middle-aged male and female mice, and report sustained PI3K inhibition without toxicity.

## 2. Materials and Methods

### 2.1. Murine Breeding and Housing Conditions

Male and female C57Bl6/J mice were bred by the AgResearch Small Animal Colony (Ruakura, New Zealand). Mice were maintained in a temperature-controlled animal facility (22 °C), with a 12-h light-dark cycle, and ad libitum access to standard chow (Teklad Global 2018, Harlan Laboratories, Madison, WI, USA), except as specified, and water. All animal experiments were approved by the University of Auckland Animal Ethics Committee (R001960).

### 2.2. Pharmacokinetics

To determine whether BYL719 can be effectively administered through diet, the pharmacokinetic profile of BYL719 administered in diet was compared with oral gavage. Twelve-week-old male C57Bl6/J mice (n = 36) were fasted overnight, following which n = 6 were killed in the fasted state and the remaining (n = 30) refed with a chow diet containing 0.3 g/kg BYL719 (MedChem Express, Monmouth Junction, NJ, USA). Following 30 min ad libitum access to a diet containing BYL719, food was replaced with standard chow and intake of BYL diet was recorded. The average ingested dose of BYL719 (3.60 mg/kg) was determined, and then a second group of mice (n = 30) was fasted overnight and administered a matched dose of BYL719 via oral gavage, with access to standard chow. Mice (n = 6 per group) were killed at 0.5, 1, 2, 6 and 24 h following diet/gavage treatment, and a further n = 6 mice were allowed ad libitum access to a chow diet contain 0.3 g/kg BYL719 for one week, after which they were killed. Mice were killed by CO_2_ inhalation, blood samples were collected via cardiac puncture and plasma was recovered by centrifuging blood samples at 6000 rpm for 5 min.

### 2.3. Plasma BYL719 Determination

Plasma samples (10 µL) were processed by adding four volumes of ice-cold acetonitrile, containing 0.1 µM ketoconazole as an internal standard, vortexed for 1 min, and centrifuged at 13,000 rpm for 5 min. The resulting supernatants were transferred to HPLC inserts and mixed 1:1 with ultrapure water (MilliQ) containing 0.1% formic acid. A BYL719 standard curve was generated by the addition of a 5 mM stock solution of BYL719 in DMSO to drug-free mouse plasma, and then serially diluted to achieve concentrations from 30 µM to 3 nM, processed as described. Quality control samples (10, 1 and 0.1 µM) were made from a separately prepared 5 mM BYL719 stock solution. BYL719 concentration was determined using an LC-MS/MS equipped with Jet Stream (Agilent 6460) following chromatographic separation in a Zorbax SB-C18 column (2.1 mm × 50 mm, 5 µm; Agilent Technologies). The mobile phase was a gradient constructed using (A) acetonitrile containing 0.1% formic acid and (B) ultrapure water containing 0.1% formic acid. The gradient profile was: 0–0.5 min, 10% A; then, this was increased to 95% by 1.5 min and maintained for 1 min, returned to 10% A over 0.5 min and held for 2 min before the next sample injection. The flow rate was maintained at 0.5 mL/min throughout the gradient condition. Injection volume was 10 μL. Column oven temperature and autosampler temperature was set to 35 °C and 4 °C, respectively. The quantitation was achieved with MS/MS detection in electrospray ionization (ESI) in positive ion mode. The instrument source parameters were set to gas temperature 300 °C; gas flow 7 L/min; nebulizer 45 psi, sheath gas temperature 300 °C, sheath gas flow 10 L/min. The ion spray voltage was set at 3000 V. Detection of the ions was carried out in multiple-reaction monitoring mode (MRM) by monitoring the transitions of *m*/*z* 442/328 as quantifier and 442/115 as qualifier for BYL719 and *m*/*z* 531/82 for ketoconazole. Quadrupoles Q1 and Q3 were set on unit resolution. To generate pharmacokinetic parameters (maximum plasma concentration, time to maximum plasma concentration, half-life, and elimination rate constant), plasma concentration versus time data from drug-fed and gavaged animals (n = 6 mice per group per time point) were analyzed by non-compartmental analysis using Phoenix WinNonlin (Certara, Princeton, NJ, USA).

### 2.4. Immunoblotting

To investigate whether the delivery of BYL719 in diet is effective in suppressing insulin signaling in older mice, experiments were initiated on 8-month-old male and female mice (n = 20 per sex). Littermates were randomized to receive a standard rodent chow diet containing either 0.3 g/kg BYL719 or 0.5% *v*/*w* DMSO as a vehicle (n = 10 per sex per diet) for 72 h, after which they were killed by cervical dislocation and liver and skeletal muscle (gastrocnemius) were rapidly dissected, snap frozen in liquid nitrogen and stored at −80 °C until analysis. Frozen tissue was weighed and homogenized in a 10-fold volume of ice-cold RIPA lysis buffer, supplemented with protease inhibitor cocktail (cOmplete mini EDTA-free protease inhibitor, Roche, Mannheim, Germany) and 2 mM PMSF before being mechanically homogenized for one minute at an oscillation frequency of 30 Hz in a Tissue Lyser II (Qiagen, Dusseldorf, Germany). Total protein concentration was determined using a BCA-protein kit (Pierce BCA Protein Assay Kit; Thermo Fisher Scientific #23225, Rockford, IL, USA) with BSA as a standard. SDS-PAGE and semi-dry transfer were used to resolve and transfer 25–30 µg protein suspended in 1× Laemmli buffer (0.5 M Tris-HCl, pH 6.8, 800 mM 2-mercaptoethanol, 2 mM EGTA, 10% glycerol, 2% SDS, 0.25% bromophenol blue) to a PVDF membrane. Following 2 h blocking in 2% fish gelatine in TBST, membranes were incubated overnight at 4 °C in primary antibodies (all from Cell Signaling Technologies; phospho Akt Ser473, #9271; total Akt, #4691; phospho insulin receptor Tyr1150/1151, #3024; total insulin receptor, #3020) diluted 1:1000 in TBST with 3% *w*/*v* BSA. Following removal of primary antibody and washing in TBST, membranes were incubated for 1–2 h in HRP-conjugated secondary antibody diluted 1:10,000 in TBST with 2% fish gelatine. Images of proteins bands were captured using a ChemiDoc™ MP Imaging System (Bio-Rad Laboratories) in the presence of chemiluminescent substrate (Clarity Western Substrate, Bio-Rad Laboratories), and bands quantified using ImageLab 5.1 software (Bio-Rad laboratories). Equal protein loading was confirmed by probing for beta actin (Sigma Aldrich, A2228) in liver, or alpha tubulin (Thermofisher Scientific, A11126) in muscle, diluted 1:5000 in TBST with 3% *w*/*v* BSA.

### 2.5. Long-Term Studies on the Effects of BYL719 in Diet

**Figure 1:**
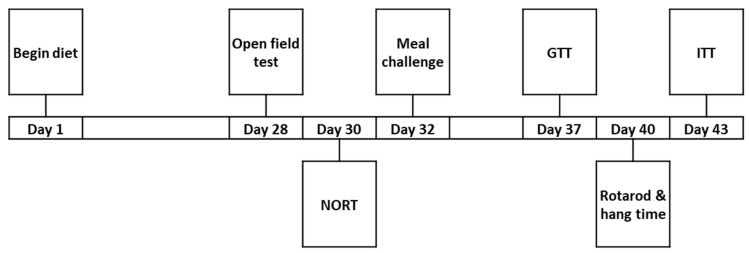
Experimental overview of long-term studies. GTT, glucose tolerance test; ITT, insulin tolerance test; NORT, novel object recognition test.

To determine whether mice develop any tolerance or toxicity in response to chronic BYL719 administration in diet, 8-month-old male (n = 12 per diet) and female (n = 15 per diet) mice were maintained on 0.3 g/kg BYL719 or vehicle diets for a period of six weeks. Body mass was monitored weekly. Beginning after three weeks of treatment with BYL719 in diet, mouse behavior, metabolic function and physical capacity were tested as follows. An overview of long-term studies is provided in Figure 1.

### 2.6. Behavioral Testing

General stress/anxiety behavior and locomotor activity was assessed using an open field (OF) test, and memory was assessed using a novel object recognition test (NORT). Both tests took place in an in-house built perspex enclosure measuring 270 mm (L) × 265 mm (W) × 350 mm (H) with enclosure walls covered to prevent external visual stimuli. For OF testing, mice were naïve to the enclosure and were placed in the center of the field. Movements were recorded for 25 min using an overhead camera (GoPro Hero 6, GoPro Inc., San Mateo, CA, USA). The enclosure area was divided into an inner and outer zone and the time spent in each zone, as well as total distance moved, was analyzed by center point tracking using EthoVision XT 12 tracking software (Noldus Information Technology, Wageningen, The Netherlands).

Following the OF test, mice were habituated with the enclosure, with NORT taking place two days after OF completion. Mice were returned to the enclosure with two identical objects place in diagonally opposite corners of the enclosure 5 cm from the wall edge. Mice were recorded for 5 min with two identical objects before being returned to their cage. After 4 h, mice were returned to the enclosure with one object the same as before, and one novel object. Mice were again recorded for 5 min, and the time spent exploring each object was determined in Ethovision XT 12.

### 2.7. Glucose Homeostasis Testing and Plasma Lipid Assessment

Glucose tolerance tests (GTT) were performed on overnight-fasted mice, and insulin tolerance tests were performed on 4 h fasted mice. Mice received an oral gavage bolus of D-glucose (2 mg/g) or intraperitoneal injection with insulin (0.6 IU/kg), and subsequently tail blood glucose was measured (Accu-chek performa; Roche, Basel, Switzerland) at the time points indicated [24]. The meal challenge experiments involved fasting mice overnight (16 h) then allowing *ad libitum* access to food for 3 h, before re-fasting for 4 h and monitoring blood glucose at baseline, after 1 and 3 h of refeeding, and then 2 and 4 h of fasting. Blood samples were collected from a tail vein in the fasted state and from fed mice after access to diet for 1 h. Plasma ALT, triglycerides and non-esterified fatty acids (NEFA) were measured by an autoanalyzer (Cobas Mira; Hoffmann-La Roche, Basel, Switzerland) and insulin with AlphaLISA immunoassay detection kit (PerkinElmer, Waltham, MA, USA). Plasma insulin and blood glucose levels were used to calculate homeostatic model assessment of insulin resistance (HOMA-IR) values using the formula from Matthews et al. [25].

### 2.8. Physical Performance Testing

Motor co-ordination was assessed by Rotarod performance. Mice were familiarized with a Rotarod (Rotamex 5, Columbus Instruments, Columbus, OH, USA), at a fixed speed of 4 RPM for 5 min, which was then increased to 6 and 8 RPM for 30 s each. This familiarization was performed twice and testing took place 48 h after the second familiarization session. For performance tests, mice were placed on the rotarod at an initial speed of 4 RPM, which increased by 0.6 RPM every 5 s until mice fell. Time and speed at which mice fell were determined using infrared sensors. The test was terminated and repeated if a mouse held onto the axle for a complete revolution without falling. Testing was repeated three times consecutively, and the best result for each mouse recorded for analysis. Grip strength was determined by inverted hang time, where mice were suspended on a wire above an open cage with bedding material. Two familiarization trials were conducted with mice suspended 20 cm and 30 cm above a cage for 30 s, prior to testing with mice suspended 30 cm above the cage. The test was terminated when mice fell or after 180 s.

### 2.9. Statistical Analyses

Statistical analyses were performed using Prism 8.0.0 (GraphPad Software Incorporated, San Diego, CA, USA), with statistical significance determined as *p* ≤ 0.05. Unless otherwise specified, results are presented as mean ± SEM. Statistical outliers were determined by ROUT test and removed prior to analysis. As indicated in the figure legends statistical analysis included independent sample student *t*-tests, one- and two-way repeated-measures ANOVA and, where appropriate, Sidak post-hoc correction for multiple comparisons was applied.

## 3. Results

### 3.1. Pharmacokinetics of Oral Gavage and Diet-Consumed BYL719

**Figure 2:**
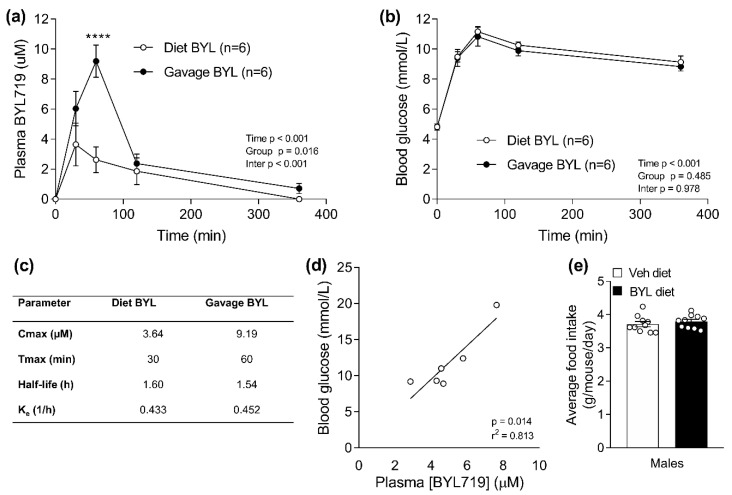
Pharmacokinetics (**a**,**b**) and changes in blood glucose (**c**) associated with delivering a single dose of BYL719 in the diet or via gavage in male mice. Plasma BYL719 (**d**) and food intake (**e**) of mice fed a control (Veh; vehicle) or BYL719 (BYL) containing diet for one week. Data are presented as mean ± SE, statistical analysis for (**a**,**b**) was by two-way repeated measures ANOVA with Sidak post-hoc, (**d**) by linear regression, and (**e**) by *t*-test. **** *p* < 0.001 vs. Veh at same time point. N = 6 per group for (**a**–**d**).

We have previously reported a dose-dependent increase in blood glucose and plasma insulin in chow-fed male mice in response to BYL being incorporated into the diet [26]. A BYL dose of 0.3 g/kg diet resulted in hyperglycemia (veh = 7.6 mM, BYL = 13.0 mM, *p* < 0.0001) and hyperinsulinemia (veh = 954 nM, BYL = 11,704 nM, *p* = 0.0003). Since orally available PI3K inhibitors are normally provided by oral gavage in rodent studies, here, we compared the pharmacokinetics of oral gavage and diet-consumed BYL. Overnight-fasted mice were provided the same dose of BYL719 (3.60 mg/kg body weight) both in diet (Diet BYL group) and by oral gavage (Gavage BYL group). Despite gavage delivery of BYL resulting in a greater peak (Figure 2a,c) and more sustained elevation in plasma BYL concentration (Figure 2a, time and interaction effect *p* < 0.001), Diet and Gavage BYL groups showed similar elevation in blood glucose for 360 min following treatment (Figure 2b, time *p* < 0.001, interaction *p* = 0.978). One week of ad libitum access to a chow diet containing 0.3 g/kg BYL resulted in an average BYL plasma concentration of 5 µM, which was greater than what was achieved in response to 1 h feeding (3.64 µM), and positively correlated with blood glucose concentration (Figure 2d). Inclusion of BYL in diet did not affect food consumption (Figure 2e).

**Figure 3:**
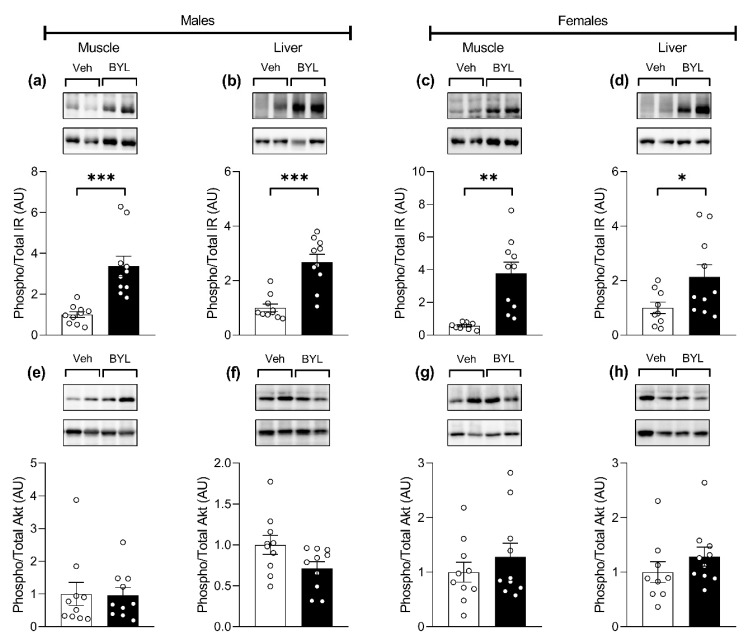
Gastrocnemius muscle and liver insulin receptor (IR) (**a**–**d**) and Akt (**e**–**h**) phosphorylation in male and female mice, following 72 h of receiving a control (Veh; vehicle) or BYL719 (BYL)-containing diet. Data are presented as mean ± SE, statistical analysis was by *t*-test, * *p* < 0.05, ** *p* < 0.01, *** *p* < 0.001 vs. Veh.

To test whether BYL719 is effective in suppressing insulin signaling (as a marker of PI3K inhibition in vivo) when delivered in the diet, we assessed the activation (phosphorylation) of the insulin receptor (IR) and Akt in insulin sensitive tissues (muscle and liver) of mice fed a BYL or Veh diet for 72 h. Both male and female BYL-treated mice showed increased hepatic and muscle (gastrocnemius) insulin receptor phosphorylation (Figure 3a–d); however, this was not translated through to Akt, with BYL groups showing similar Akt phosphorylation in muscle and liver (Figure 3e–h).

### 3.2. Six Weeks of BYL719 Impairs Glucose Tolerance without Affecting Lipid Homeostasis or Toxicity Measures

**Figure 4:**
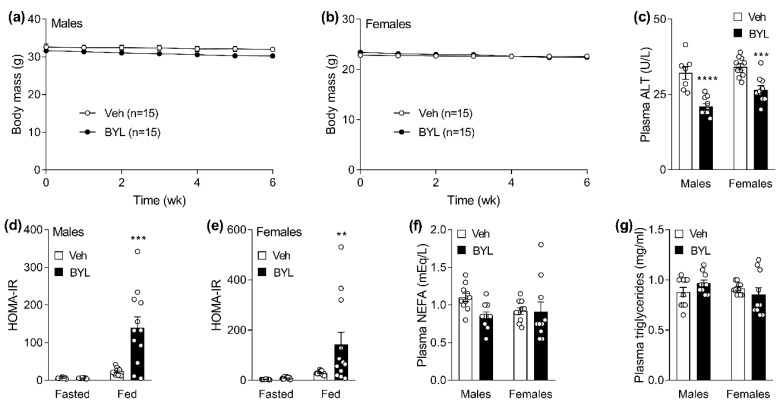
Body mass (**a**,**b**), plasma alanine aminotransferase (ALT) (**c**), HOMA-IR (**d**,**e**), and plasma non-esterified fatty acids (NEFA) (**f**) and triglycerides (**g**) of male and female middle-aged mice following 6 weeks of receiving a control- (Veh; vehicle) or BYL719 (BYL)-containing diet. Data are presented as mean ± SE, statistical analysis for was by two-way repeated measures ANOVA (**a**,**b**) and two-way ANOVA (**d**,**e**) with a Sidak post-hoc and/or *t*-test (**c**,**f**,**g**). ** *p* < 0.01, *** *p* < 0.001, **** *p* < 0.0001 vs. Veh, n = 6 per group for (**a**–**d**).

Having established the pharmacokinetics of BYL719 when delivered in the diet and having shown evidence that this delivery method impaired insulin signaling in insulin sensitive tissues, we next assessed the effect of prolonged (6 weeks) BYL diet exposure on glucose and lipid homeostasis as well as markers of toxicity (change in body mass and liver enzyme ALT). Body weight was not significantly different between BYL- and Veh-treated mice, though it did trend towards decreasing over time in BYL-treated mice (*p* = 0.1 in males, *p* = 0.08 in females, Figure 4a,b). The liver damage marker ALT was lower in plasma in both male and female mice (Figure 4c), indicating no toxicity of consuming BYL in the diet ad libitum. Homeostatic model assessment of insulin resistance (HOMA-IR) is a method used to estimate whole body insulin resistance based on the relationship between plasma glucose and insulin levels [25]. Interestingly, following an overnight fast, both male and female Veh- and BYL-treated mice had a similar HOMA-IR index. While in the fed state, both male and female mice show evidence of insulin resistance (higher HOMA-IR; Figure 4d,e), which was independent of any major disruptions in lipid homeostasis (as assessed by plasma NEFA and triglycerides; Figure 4f,g).

**Figure 5:**
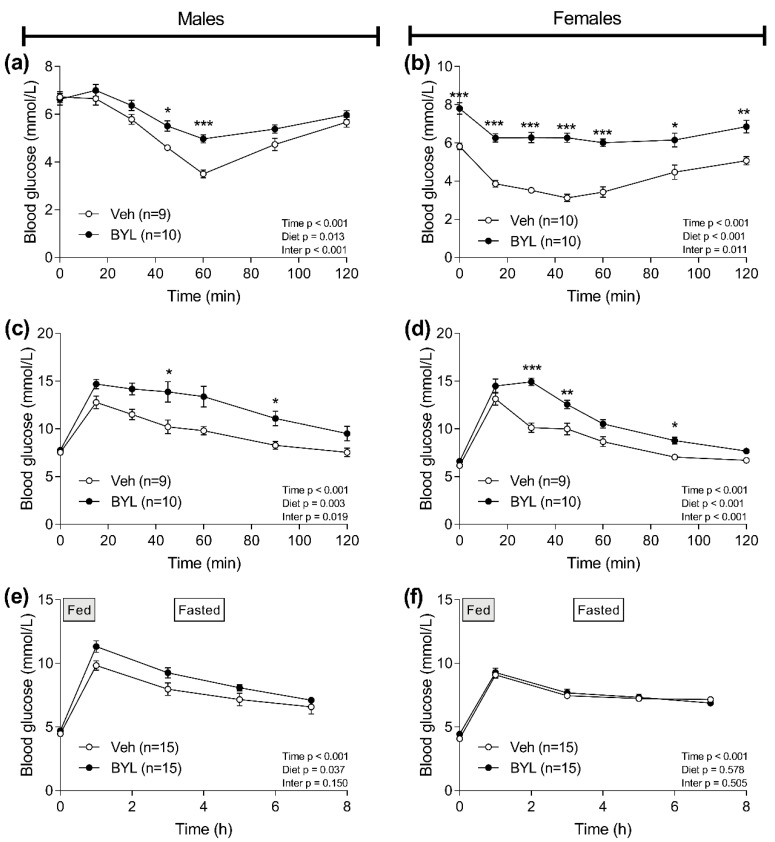
Glucose (**a**,**b**) and insulin tolerance tests (**c**,**d**), and blood glucose response to a meal challenge (**e**,**f**) of male and female middle-aged mice following 6 weeks of receiving a control- (Veh; vehicle) or BYL719 (BYL)-containing diet. Data are presented as mean ± SE, statistical analysis was by two-way repeated measures ANOVA with Sidak post-hoc. * *p* < 0.05, ** *p* < 0.01, *** *p* < 0.001 vs. Veh.

To further explore the effect of 6 weeks of BYL-diet feeding on glucose homeostasis, we undertook insulin and glucose tolerance tests in BYL- and Veh-treated mice. Both male and female BYL-treated mice showed impaired ability to lower blood glucose in response to an i.p. dose of insulin (Figure 5a,b, time × diet interaction *p* < 0.05) and had a higher and more prolonged elevation of blood glucose following an oral bolus of glucose (Figure 5c,d). While these data indicate marked insulin resistance, suggesting even after 6 weeks of ad libitum BYL-diet treatment BYL was still effective in suppressing insulin signaling, we next assessed the ability to lower blood glucose following a more physiological meal challenge. Despite consuming the same amount of food during the meal challenge, male BYL mice had overall mildly elevated blood glucose (diet effect *p* = 0.037), but the effect of the meal on blood glucose levels was not altered by BYL treatment in male (interaction *p* = 0.15; Figure 5e). In contrast, female BYL-treated mice showed a similar blood glucose response to a meal as Veh-treated mice (Figure 5f).

### 3.3. Six Weeks of BYL719 Does Not Affect Markers of Muscle Function or Behaviour

**Figure 6:**
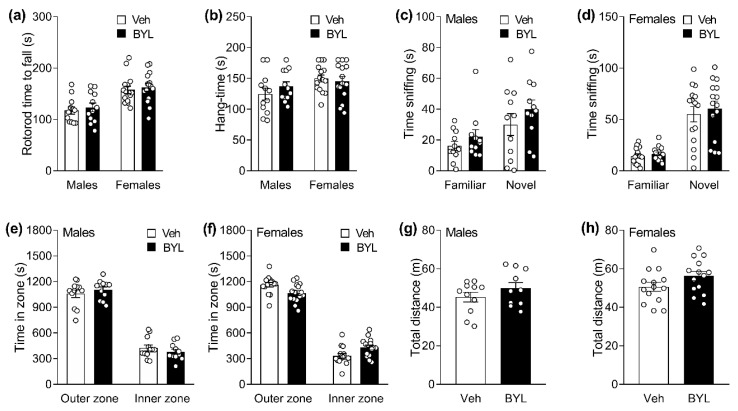
Rotarod (**a**) and hang time performance (**b**), novel and familiar object sniffing time (novel object test, **c**,**d**), time spent in inner and outer zone (**e**,**f**) and total movement distance (**g**,**h**) during open field test for middle-aged male and female mice after receiving a control- (Veh; vehicle) or BYL719 (BYL)-containing diet. Data are presented as mean ± SE, statistical analysis was by *t*-test (**a**,**b**,**g**,**h**) or two-way ANOVA with Sidak post-hoc (**c**–**f**).

We next assessed the effect of prolonged BYL or Veh treatment on rotarod performance as a measure of coordination, and hang time as a marker of muscle strength, in male and female mice. BYL treatment did not affect fall time on a rotarod (Figure 6a) or hang time (Figure 6b) in either male of female mice. Similarly, BYL did not affect object exploration in a novel object test (Figure 6c,d), nor locomotion or time spent in the inner or outer zone of an open-field test (Figure 6e–h).

## 4. Discussion

The PI3K p110α inhibitor BYL719 (alpelisib) is currently an approved cancer therapeutic; however, genetic studies in rodents suggest that long-term suppression of insulin/IGF1 signaling mediated PI3K p110α can extend life and healthspan of aged mice. As a preliminary investigation to develop a model for investigating long-term pharmacological suppression of PI3K p110α signaling in aging, we first assessed the pharmacokinetics of delivering BYL719 to mice in diet, and then the ability of BYL719 to suppress insulin signaling when provided in the diet of middle-aged mice for an extended (6 weeks) period. We report that when consumed in the diet, a single dose of BYL719 has a lower Cmax, but similar half-life as compared to delivery by oral gavage. Although treatment was non-toxic, males and females do not fully adapt to BYL719, with insulin resistance and glucose intolerance being evident following 6 weeks of treatment. These data suggest that supplementing rodent diet with BYL719 is a viable approach to study the long-term effects of pharmacological suppression of PI3K p110α on aging and metabolic function.

Supplementation of diet or drinking water is a common approach for drug delivery in long-term rodent studies [21,22,23]. This mode of drug delivery pharmacokinetics is important to consider when translating to a single bolus once or twice daily dosing, which is a common approach clinically. Interestingly, when the same dose of BYL719 was consumed in a single bolus (gavage) or over a 1 h period in food this led to a similar increase in blood glucose (a marker of PI3K inhibition), which reflected both methods having a similar half-life and elimination rate, indicating efficacy of diet delivery. The plasma half-life determined here is shorter than the half-life reported for BYL719 in other rodent studies [27,28]; however, the Cmax achieved here is greater than that of 0.52 µM reported by Furet et al. [28] using a comparable dose of 3 mg/kg. However, this could be due to differences in specific strains and/or species used, as to our knowledge, this is the first assessment of pharmacokinetic parameters of BYL719 in C57Bl6/J mice.

Insulin acts via the insulin receptor-PI3K-Akt signaling pathway in skeletal muscle and adipose tissue to promote glucose disposal, and in the liver to suppresses endogenous hepatic production [29]. Efficacy of providing BYL719 in diet was further supported by its ability to prevent increases in Akt phosphorylation in peripheral tissues (muscle, liver, fat) despite marked increases in insulin receptor activation (phosphorylation), indicating effective inhibition of PI3K. The elevated HOMA-IR in the BYL719-treated group in the fed state suggests that for a given level of insulin BYL719 mice are less able to lower blood glucose and would be consistent with the observation of greater insulin receptor phosphorylation without translating, via downstream (PI3K-mediated) signaling, to lower blood glucose levels.

Rodents generally do not consume food as a large bolus but feed regularly throughout the dark cycle, and therefore, it was important to assess the plasma level of BYL719 when consumed normally in diet. The plasma levels in the early (4 h) light cycle following one week of BYL719 diet feeding were slightly higher than the peak achieved during pharmacokinetics assay. Given the half-life of ~1.6 h this potentially indicates accumulation of BYL719 with continual feeding, which may better model dosing in humans with a single 400 mg oral dose having the longer elimination half-life of 13.7 h [30]. The circulating concentrations achieved with the incorporation of BYL719 into the diet impair both glucose tolerance and insulin sensitivity (as assessed by glucose and insulin tolerance tests) for at least 6 weeks in both male and female mice, suggesting maintained PI3K inhibition. This observation is somewhat in contrast with our previous data showing following 20 days i.p. treatment of young (5 weeks) mice with A66, a PI3K p110α inhibitor, did not suppress insulin induced reductions blood glucose levels [18]. While this could reflect the differences in once daily vs. continued diet dosing, mice with genetic suppression of PI3K signaling through heterozygous mutation of p110α to a kinase-dead D933A allele, or overexpression of PTEN, have normal or improved responses to a glucose bolus. This perhaps suggests that when PI3K inhibition is initiated during development/early life and this promotes adaptions to maintain glucose homeostasis [9,13]. Interestingly, a mild level of impaired glucose metabolism is a feature of several genetic and pharmacological murine models that target the insulin signaling pathway to extend lifespan, and mild insulin resistance with proposed to be a protective mechanism during aging [10,31,32]. It has been suggested that partial suppression inducing adaptation responses that mimic calorie or carbohydrate restriction, while complete ablation impaired cellular function [24,33]. Indeed, complete adult-induced ablation of the peripheral insulin receptor in adult mice reduces lifespan [34] and germline ablation of insulin receptor or PI3K is embryonically lethal or can cause death at a young age [2]. Thus, a limitation of this study was that only one dose of BYL719 (0.3 g/kg) diet was used, and this was based on previous dose-response data [26] indicating a partial impairment of insulin signaling and modulating this dose may affect the efficacy of the treatment.

Previously, 10-week treatment of adult chow-fed mice with a more broad-spectrum PI3K inhibitor (CNIO-PI3Ki, which inhibits PI3Kα, PI3Kδ and PI3Kγ) did not affect fasted blood glucose levels of chow diet fed mice, while reducing fat mass and lowering blood glucose in murine obesity models [14]. Somewhat consistent with these findings, BYL719 did not affect overnight-fasted blood glucose, and in response to a meal test in female BYL719 treated mice showed normal glucose tolerance and males glucose tolerance was only mildly impaired. This suggests that complete PI3K activation is not required to maintain glucose homeostasis, and indeed, adipose tissue specific deletion can protect from diet-induced metabolic dysfunction [35], while muscle-specific deletion can enhance mitochondrial biogenesis [36]. The difference in response of males and females to the meal test after four weeks of BYL in diet may be explained by the effects of estradiol, which increases insulin sensitivity [37] and protects against diet-induced obesity and glucose intolerance [38]. Sexual dimorphic effects of PI3K inhibition have been reported previously, with partial inactivation of PI3K only extending lifespan in male mice [9].

Diet supplementation with BYL719 did not appear to adversely impact overall health of mice, indicated by no substantial effect on body weight, food intake, strength/coordination, and reduced circulating levels of the liver damage marker ALT. While the PI3K signaling pathway has been implicated in age-associated cognitive impairment [39], BYL719 does not cross the blood brain barrier, and thus, it is not surprising that in middle-aged mice BYL719 treatment did not affect mouse exploratory activity. With age-related cognitive decline, exploratory activities and muscle performance decrease; therefore, in longer-term studies it will be important to assess whether pharmacological PI3K inhibition can attenuate these markers of aging.

## 5. Conclusions

Taken together, our results demonstrate that supplementing BYL719 in the diet of mice has a similar pharmacokinetic profile to gavage administration, is non-toxic and maintains its ability to impair glucose homeostasis for at least six weeks. This suggesting that delivering BYL719 in the diet is an appropriate approach to assess the effect of long-term pharmacological PI3K inhibition on aging and metabolic function.

## Data Availability

Data is contained within the article and raw data can be provided upon request.
